# Film measurements of kilovoltage x-ray percentage depth doses for different energies and field sizes

**DOI:** 10.1093/bjr/tqaf107

**Published:** 2025-05-27

**Authors:** Tze Yee Lim, Dershan Luo, Jamshid Moradi-Kurdestany, Xin Wang, Ramesh Tailor

**Affiliations:** Department of Radiation Physics, The University of Texas MD Anderson Cancer Center, Houston, TX 77030, United States; Department of Radiation Physics, The University of Texas MD Anderson Cancer Center, Houston, TX 77030, United States; Department of Radiation Oncology, Mercy Coletta Cancer Center, Oklahoma City, OK 73120, United States; Department of Radiation Physics, The University of Texas MD Anderson Cancer Center, Houston, TX 77030, United States; Department of Radiation Physics, The University of Texas MD Anderson Cancer Center, Houston, TX 77030, United States

**Keywords:** kilovoltage x-ray, superficial, orthovoltage, percentage depth dose

## Abstract

**Objectives:**

Kilovoltage x-ray therapy, which includes superficial x-rays and orthovoltage x-rays, is particularly suited for treatments near the skin surface. Radiation dose deposition at depth is typically characterized by percentage depth dose (PDD) curves. However, PDD data are difficult to obtain in kilovoltage x-ray therapy, hence clinics often rely on *British Journal of Radiology* Supplement 25 (BJR-25) archival data. This study measured PDD for different energies and field sizes on the Xstrahl 300 kilovoltage x-ray therapy unit for comparison with BJR-25 data.

**Methods:**

We irradiated EBT3 film in water using different energy and field size combinations, then, compared the acquired PDD curves with BJR-25 data. We used 75, 125, and 250 kVp beams, for 10 × 10 cm^2^ open field, 4 × 4 cm^2^ closed-ended square applicator, and open-ended circular applicators of 1.5, 2.0, 2.8, and 4.7 cm diameter.

**Results:**

PDD for different energies and field sizes on the Xstrahl 300 kilovoltage x-ray therapy unit agreed well with BJR-25 data. The average difference between measured and BJR-25 data was −0.3 ± 1.8%; 85.9% of the individual local differences were within ±3%, 94.8% within ±4%, and 99.5% within ±5%.

**Conclusions:**

The PDD curves presented here, together with BJR-25 data, can serve as useful comparison datasets for the commissioning of kilovoltage x-ray machines.

**Advances in knowledge:**

Our film-based measurement technique provides high spatial resolution PDD datapoints for a modern kilovoltage x-ray unit, adding to the limited published data in the kilovoltage energy range.

## Introduction

Radiotherapy has many dermatologic indications and is frequently administered for nonmelanoma skin cancer,^1^ which is the fifth most prevalent cancer globally.^2^ Nonmelanoma skin cancer has been treated with kilovoltage x-ray therapy, brachytherapy, megavoltage electron therapy, and megavoltage photon therapy.^3^^,^^4^ Kilovoltage x-ray therapy, encompassing superficial x-ray and orthovoltage x-ray therapy, deposits its maximum dose at the skin, which makes this modality particularly suited for superficial targets (eg, nonmelanoma skin cancer).^3^^,^^5^ Patient-specific and tumour-specific characteristics are important to consider when choosing the treatment modality,^5^^,^^6^ and appropriate use criteria^7^ can aid patient and tumour selection. Compared with other more invasive treatment options, kilovoltage x-ray therapy can provide therapeutic efficacy as well as excellent cosmetic and functional outcomes.^4^^,^^5^^,^^8^

The percentage depth dose (PDD) describes the dose (expressed as a percentage) deposited at a particular depth relative to the maximum dose (100%), which occurs at the surface for kilovoltage x-ray beams. The main reference for kilovoltage x-ray PDD data is the *British Journal of Radiology* Supplement 25 report (BJR-25),^9^ published in 1996. PDD measurements for kilovoltage x-ray beams can be difficult to obtain, especially at and near the surface. The energy dependence of detectors necessitating field size and depth correction factors, and the size of detectors hampering accurate surface measurements, are among the challenges of kilovoltage PDD measurements.^10–16^ Due to these challenges, clinical practice often relies on BJR-25 data.^12^ Although BJR-25 data are widely used,^17^ consensus is limited on the continued validity of the data.^11^^,^^13^^,^^16^^,^^18–21^

The purpose of the current study was to characterize the PDD on an Xstrahl 300 kilovoltage x-ray therapy unit for different energies and field sizes, as well as assess the validity of BJR-25 data. To do this, we irradiated film in water using different energy and field size combinations. The PDD curves presented here can guide physicians in their selection of energy and field size appropriate for a patient’s disease and may serve as a useful comparison dataset for others with kilovoltage x-ray machines.

## Methods

We performed our measurements on the Xstrahl 300 kilovoltage x-ray therapy unit (Xstrahl Inc, Suwanee, GA, USA) using three beam energies: 75 kVp (2.2 mm Al half-value layer [HVL]), 125 kVp (3.5 mm Al HVL), and 250 kVp (1.3 mm Cu HVL). For each energy, we investigated six field sizes: 10 × 10 cm^2^ open field, 4 × 4 cm^2^ closed-ended square applicator, and open-ended cylindrical applicators of 1.5, 2.0, 2.8, and 4.7 cm diameter. The 10 × 10 cm^2^ open field was achieved using an Xstrahl insert that produces a 10 × 10 cm^2^ field at 50 cm focus-to-surface distance (FSD) and is part of a high-reproducibility measurement system for monthly and annual output checks.^22^ The circular applicators originally came with our previous kilovoltage x-ray unit (RT-250; Philips Medical System, Inc, Cleveland, OH, USA). In total, we investigated 18 different energy and field size combinations.

The AAPM TG-61 protocol^10^ noted that a well-designed cylindrical chamber may be a suitable detector for PDD measurements, as it has a nearly flat energy dependence between 40 and 300 kV, but the report also noted that the chamber’s measurement depth is limited to its outer radius. We measured PDD in water using Gafchromic EBT3 film (Ashland Inc., Wilmington, DE, USA), chosen due to its high spatial resolution, waterproof nature, minimal sensitivity to ambient light, ease of processing, and minimal energy dependence.^23–25^ Because film response can vary by batch,^26^ we used a single batch of film for the current study. Before the PDD measurements, film calibration curves were obtained for each energy (detailed methodology described elsewhere).^27^

**Figure 1.**
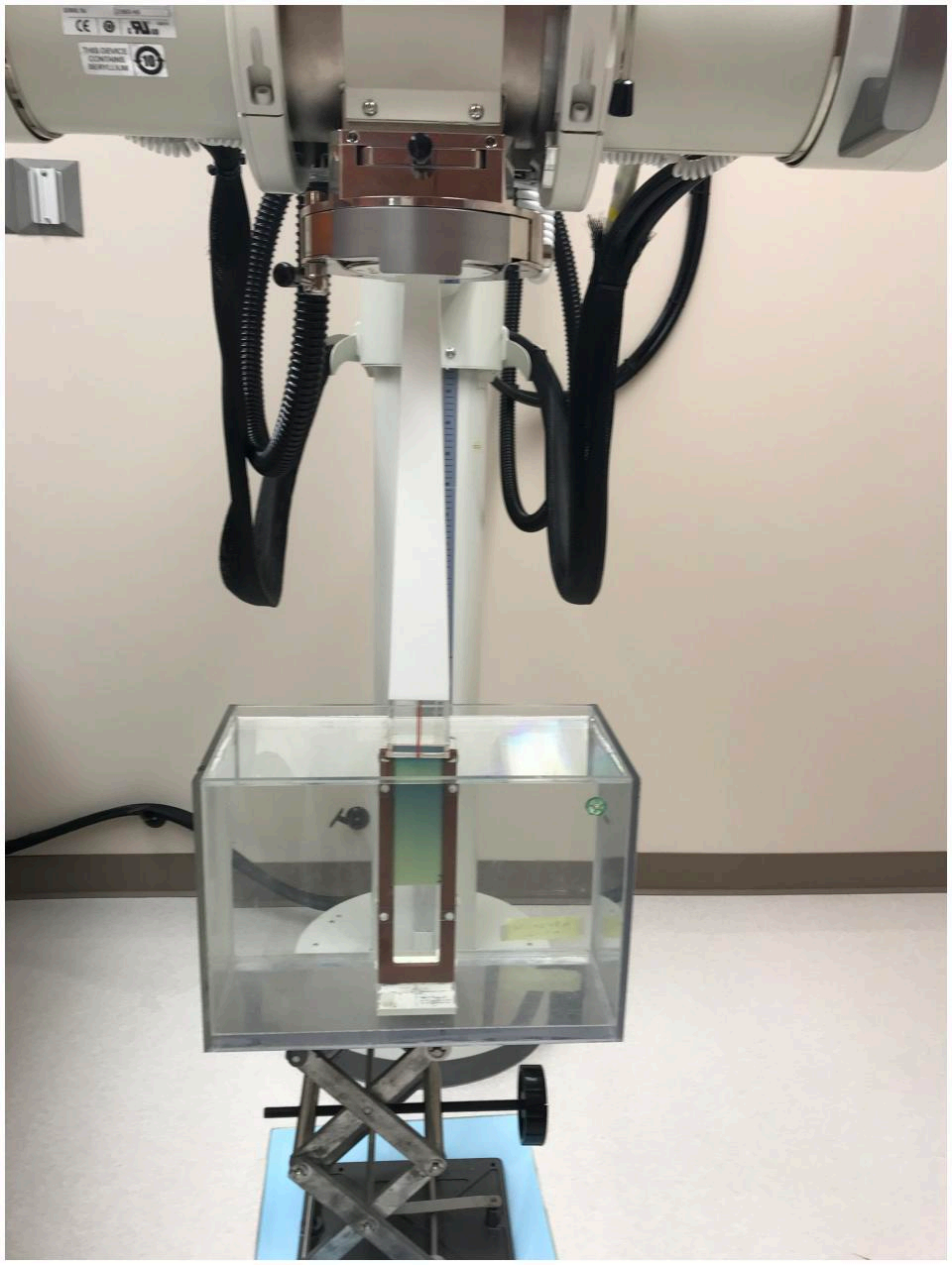
Setup for percentage depth dose measurements with film in water on the Xstrahl 300 kilovoltage x-ray therapy unit.

The experimental setup is shown in Figure 1. A water tank was placed atop foam blocks and a scissor stand. A film stand^28^ was screwed to the bottom of the water tank. The film stand was centred along the beam axis of the Xstrahl 300 kilovoltage x-ray therapy unit using a crosshair device.^22^ To alleviate the increase in beam attenuation by the film relative to water, we tilted the film by 2° with respect to the beam’s central axis^29^ using a plumb line.^28^ The FSD was 27.1 cm for the circular applicators and 50 cm for the square fields. For the applicator measurements, the applicator openings were set to the water surface. For the open field measurements, a mechanical pointer^22^ was used to set the FSD to 50 cm. Water was slowly added into the tank until filled to about 0.5 mm above the film’s top edge. By looking through the water to observe the gap between the film’s top edge and its reflection, we incrementally removed the water using a large syringe to reduce the gap to zero, thereby aligning the film’s edge to the water surface.

**Figure 2.**
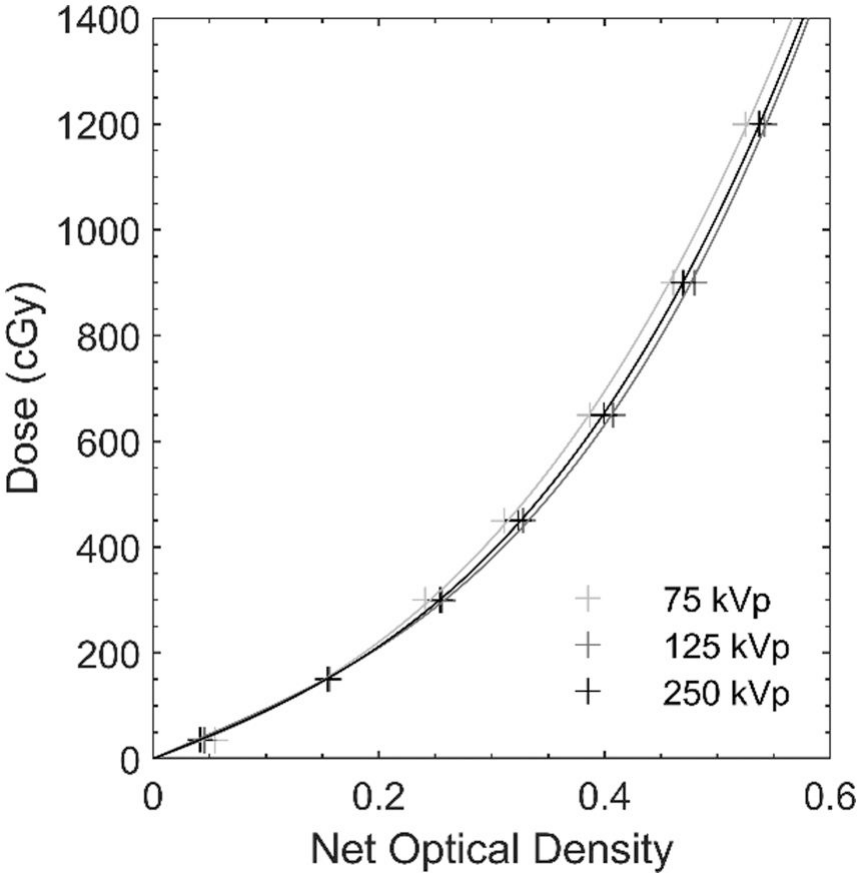
Energy-specific film calibration curves to convert net optical density to dose.

For the PDD measurements, 37 film pieces were cut and marked to ensure the same orientation during scanning. Before irradiation, each film piece was scanned to obtain its unexposed value. An unirradiated film piece was also preserved for background correction. For each of the 18 energy and field size combinations, two film pieces were irradiated with the setup described above. Immediately after irradiation, films were wiped to reduce humidity effects on the net optical density, as well as to remove fingerprint marks due to handling. Approximately 24 h after irradiation, all film pieces were scanned using an Epson Expression 10000XL flatbed scanner (Epson America, Inc, Los Alamitos, CA, USA). To position each film piece at the centre of the scanner bed reproducibly, we placed the film pieces within a cardboard cutout template. Prior to scanning, a 3 mm-thick polycarbonate sheet was placed on top of the film to ensure film flatness. Each film piece was repeatedly scanned three times in transmission mode, with 48-bit RGB colour mode, 72 dpi resolution, and no image correction, and files were stored as TIFF images. Image analysis was performed using ImageJ (National Institutes of Health, Bethesda, MD, USA). A thin rectangular region-of-interest was defined at midline along the film’s long axis (beam direction). In this region-of-interest, we recorded the mean pixel intensities from the red channel, which has the maximum sensitivity. Net optical density was then calculated^30^ and converted to dose using the previously generated energy-specific calibration curves (Figure 2). This was repeated for each of the two films per energy and three scans per film, and then, the average was taken. The average doses were plotted against depth (correcting for intentional 2° tilt). Due to the high spatial resolution, no lines were needed to connect the points and no smoothing was performed.

For comparison with our measurements, BJR-25 data were interpolated to match our beams’ HVL, correcting for FSD and field size. The measured PDD and BJR-25 data were then plotted together, and differences were calculated. Furthermore, the depth of penetration at various clinically relevant dose levels, R_90_ (depth of 90% of maximum dose), R_80_ (depth of 80% of maximum dose), and R_50_ (depth of 50% of maximum dose), were presented. The measured PDDs using a 4 × 4 cm^2^ square applicator for the investigated kilovoltage photon energies were also compared with institutional data for megavoltage electron energies (6, 9, 12, 16, and 20 MeV).

## Results

**Figure 3.**
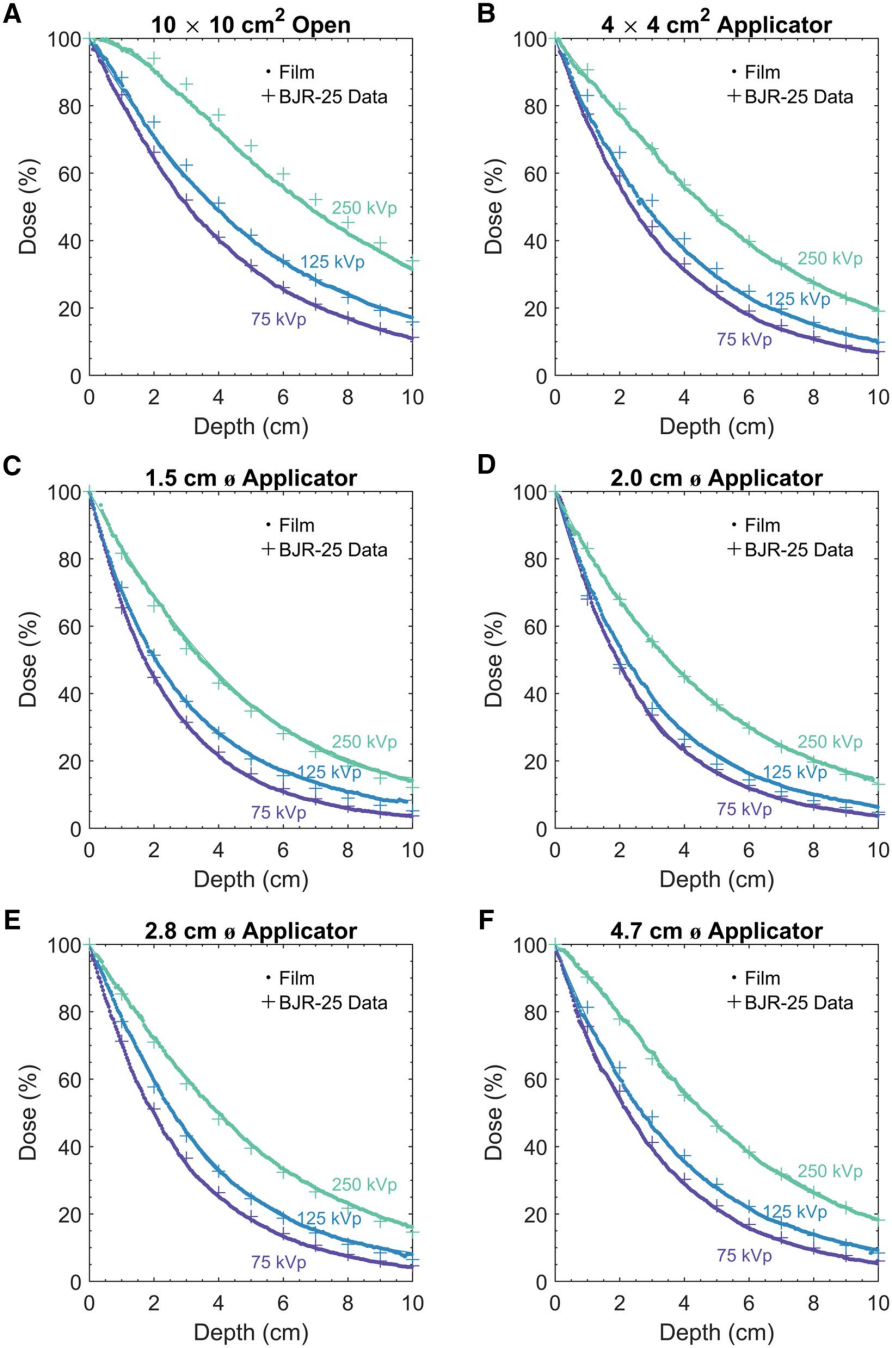
Percentage depth dose curves for 75 kVp (2.2 mm Al HVL), 125 kVp (3.5 mm Al HVL), and 250 kVp (1.3 mm Cu HVL) beams based on film measurements and compared with BJR-25 data, grouped by field sizes: (A) 10 × 10 cm^2^ open (no applicator), (B) 4 × 4 cm^2^ square applicator, (C) 1.5 cm diameter circular applicator, (D) 2.0 cm diameter circular applicator, (E) 2.8 cm diameter circular applicator, and (F) 4.7 cm diameter circular applicator.

Figure 3 shows the PDD for 75, 125, and 250 kVp, grouped by field size (10 × 10 cm^2^ open field, 4 × 4 cm^2^ square applicator, and 1.5, 2.0, 2.8, and 4.7 cm diameter circular applicators). The PDD could be fitted by third- and fourth-order polynomials (Table 1). For a given applicator size, chosen based on disease lateral extent, the PDD of varying energies can be examined to select the appropriate energy. Optimal energy selection leads to better patient outcomes.^8^ In the kilovoltage x-ray energy range, most of the dose is deposited near the surface. As energy increases, the dose falloff with depth becomes less steep. Higher energies allow for greater dose deposition near the surface to cover the full thickness of the lesion, but higher energies also deposit greater dose at deeper depths where there may be organs at risk. Conversely, lower energies deposit less dose at deeper depths, but there might not be enough dose at shallow depths to cover the full thickness of the lesion. Nevertheless, because there is still significant dose at greater depths (eg, beyond 5 cm), the beam should be placed to avoid other parts of the body downstream, if possible.

**Table 1.** Polynomials of the fits to the PDD data for 75, 125, and 250 kVp with various field sizes.

Energy	Field size	D = A_4_d^4^ + A_3_d^3^ + A_2_d^2^ + A_1_d + 1			
		A_4_	A_3_	A_2_	A_1_
75 kVp	10 × 10 cm^2^ open	0	−0.0006	0.01843	−0.2131
	4 × 4 cm^2^	6.03E−05	−0.00231	0.03608	−0.2831
	4.7 cm ⌀	0.000116	−0.00357	0.04587	−0.3113
	2.8 cm ⌀	0.000144	−0.00446	0.05527	−0.3463
	2.0 cm ⌀	6.95E−05	−0.00305	0.04787	−0.3396
	1.5 cm ⌀	0.000209	−0.00607	0.0693	−0.3905
125 kVp	10 × 10 cm^2^	−3.8E−05	0.000557	0.005633	−0.1567
	4 × 4 cm^2^	3.24E−05	−0.00146	0.02658	−0.2417
	4.7 cm ⌀	1.41E−05	−0.00114	0.02565	−0.2467
	2.8 cm ⌀	−5.2E−06	−0.00068	0.0227	−0.2455
	2.0 cm ⌀	4.8E−05	−0.00224	0.03812	−0.2983
	1.5 cm ⌀	0.000191	−0.00538	0.06016	−0.3465
250 kVp	10 × 10 cm^2^	−9.3E−05	0.002665	−0.02281	−0.01293
	4 × 4 cm^2^	−4.2E−05	0.000999	−0.00258	−0.1125
	4.7 cm ⌀	−7.9E−05	0.002035	−0.01162	−0.08934
	2.8 cm ⌀	−4.9E−05	0.000861	0.002532	−0.1465
	2.0 cm ⌀	0	−0.0005	0.01541	−0.1909
	1.5 cm ⌀	0	−0.00013	0.01019	−0.1739

Comparison of our film measurements with BJR-25 data (Figure 3) showed reasonable agreement overall. For all energies and field sizes, the average difference was −0.3 ± 1.8%; 85.9% of the individual local differences between measured and BJR-25 data were within ±3%, 94.8% within ±4%, and 99.5% within ±5%. The range of the local differences between the measurements and BJR data was [−3.1%, +2.5%] for 75 kVp, [−5.0%, +5.3%] for 125 kVp, and [−4.4%, +3.3%] for 250 kVp. The average difference (for each energy and field size combination) was [−1.5%, +0.0%] for 75 kVp, [−2.4%, +2.8%] for 125 kVp, and [−3.7%, +2.1%] for 250 kVp.

**Figure 4.**
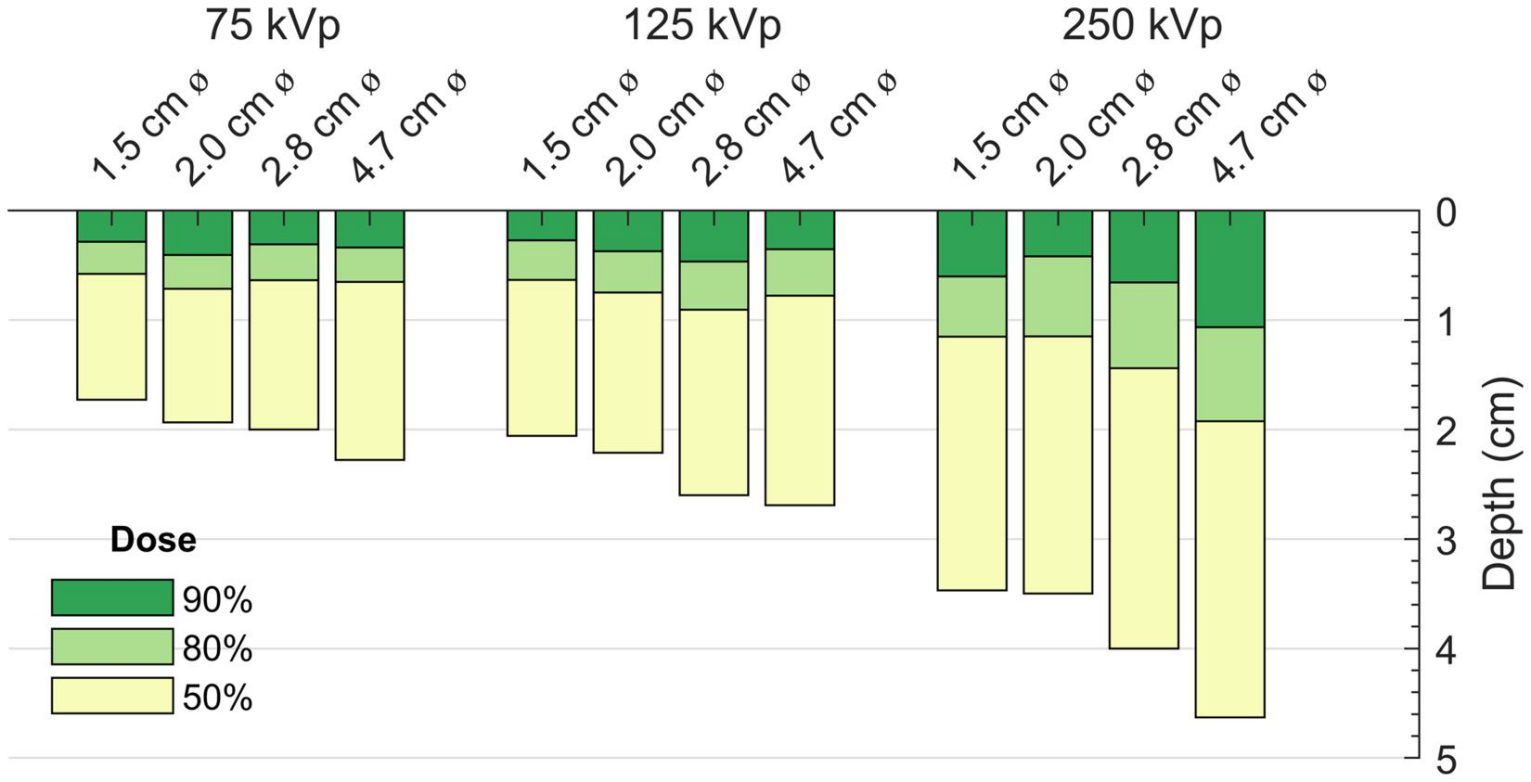
Depth of dose penetration at various dose levels, 90% of the maximum dose (dark green), 80% of the maximum dose (light green), and 50% of the maximum dose (yellow), for 75 kVp (2.2 mm Al HVL), 125 kVp (3.5 mm Al HVL), and 250 kVp (1.3 mm Cu HVL) beams, using circular applicators of diameters 1.5 cm, 2.0 cm, 2.8 cm, and 4.7 cm.

Figure 4 shows the depth of penetration for various field sizes, grouped by energy. For a given energy, larger field sizes increased penetration (but to a lesser extent compared with increasing energy to increase penetration). The magnitude of augmented penetration with field size was more prominent for higher energies.

**Figure 5.**
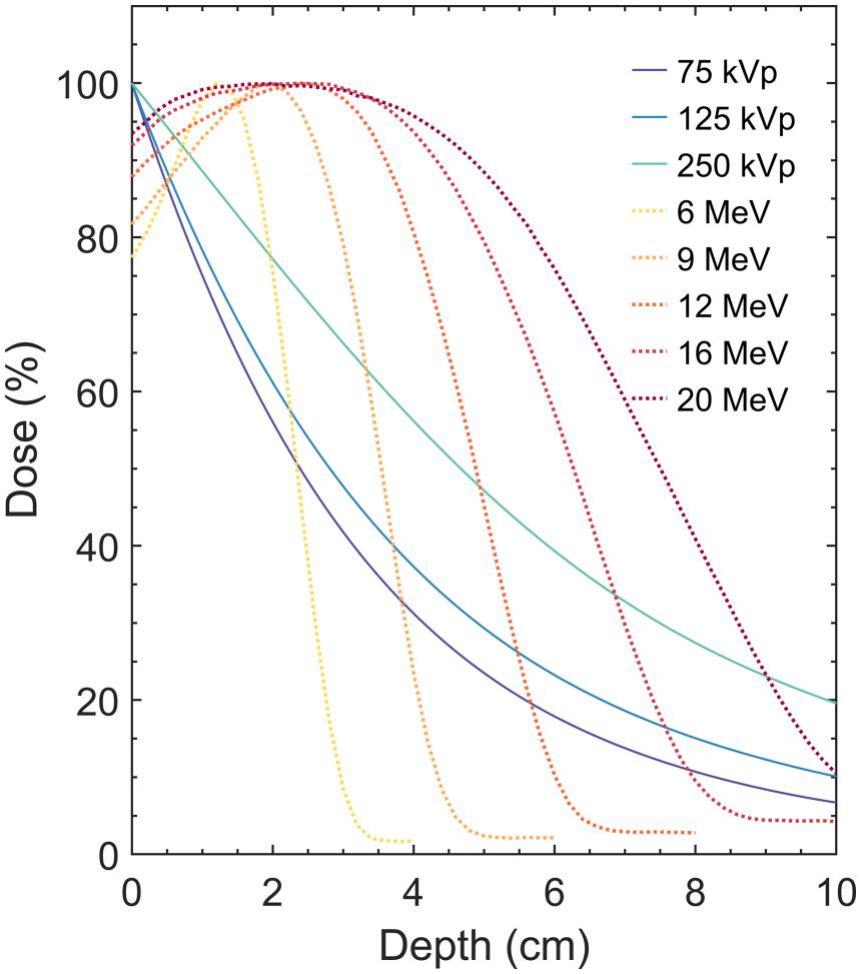
Comparison of percentage depth doses for kilovoltage photon energies (75, 125, and 250 kVp) with those of megavoltage electron energies (6, 9, 12, 16, and 20 MeV) using a 4 × 4 cm^2^ square applicator.

Figure 5 shows the comparison of the measured PDD of kilovoltage photon beams with that of institutional data on electron beams, another radiotherapy modality used for shallow diseases. Megavoltage electron beams have a buildup region and much greater penetration depths compared with kilovoltage photon beams (Figure 5). Therefore, for very superficial lesions, megavoltage electron beams may not be suitable (unless a bolus is used). However, compared with the gradual dose falloff of kilovoltage photon beams, electron beams fall off rapidly to a negligible dose, resulting in less dose to deep organs at risk.

## Discussion

We found that the PDD on an Xstrahl 300 kilovoltage x-ray therapy unit at different energies and field sizes was consistent with BJR-25 data.

On the lower-energy end of superficial x-rays (<100 kVp), the difference in local PDD between measured and BJR-25 data was within [−3.1%, +2.5%] for the 75 kVp beam, which is similar to findings reported by Jurado et al^21^ (within −4% for 50 kVp, [−5%,+3%] for 80 kVp), Hill et al^20^ (<4% for 50 kVp), Evans et al^12^ (up to −5.6% for 70 kVp), and Xiong et al^31^ (within 6% from figures of representative PDD for <100 kVp). However, our findings differ from those of Prentou et al^13^ (>10% for 40 kVp), Aspradakis and Zucchetti^19^ (>10% for 50-100 kVp), and Hill et al^20^ (up to 12.5% for 75 kVp). Prentou et al^13^ presented findings based on Monte Carlo simulations rather than physical measurements, and for a lower-energy beam, which may contribute to the disagreement. Aspradakis et al^19^ obtained measurements using ionization chambers with relatively big volumes or in a water-equivalent plastic phantom instead of water, thereby increasing measurement uncertainty. BJR-25 data also have some uncertainty, which Hill et al^20^ postulated was the source of their observed discrepancy. Our film measurements showed average differences of −1.5% to 0.0% for 75 kVp, similar to that reported by Xiong et al^31^: [−3.6%, +0.4%] using solid-state detectors and [−2.6%, +3.0%] using a PinPoint ionization chamber for <100 kVp beams.

On the higher-energy end of superficial x-rays (100-150 kVp), the difference in local PDD between measured and BJR-25 data was within [−5.0%, +5.3%] for the 125 kVp beam, which is consistent with reports by Xiong et al^31^ (within 6% from figures of representative PDD for ≥100 kVp), Li et al^16^ (within 2% for 100 kVp), Hill et al^20^ (<1% for 100 kVp), Prentou et al^13^ (up to 3.5% for 100 kVp), Evans et al^12^ (up to −2.6% for 100 kVp), and Aspradakis and Zucchetti^19^ (within ±5% for ≥100 kVp). Nonetheless, our findings disagree with Johnstone et al,^18^ who reported large local differences of [−14.0%, +15.7%], with an overall average difference of 4.9 ± 2.1% for their measurements using 80, 120, and 140 kVp beams, whereas our average difference was 0.0 ± 2.3% for 125 kVp. Johnstone et al^18^ obtained their measurements using relatively large-volume detectors and compared their findings with BJR-25 data to the nearest matching HVL rather than interpolating to beam-specific HVL, which may account for the discrepancy. Our film measurements showed an average difference of [−2.4%, +2.8%] for 125 kVp, similar to that reported by Xiong et al^31^: [−3.7%, +1.4%] using solid-state detectors and [−1.3%, +3.7%] using a PinPoint ionization chamber for ≥100 kVp beams.

For the orthovoltage x-rays (150-500 kVp), our findings showed that the difference in local PDD between measured and BJR-25 data was within [−4.4%, +3.3%] for the 250 kVp beam, similar to that reported by Evans et al^12^ (up to +6.2% for 180 kVp and up to −2.4% for 250 kVp), Prentou et al^13^ (up to 2% for 180 kVp and up to 3% for 220 kVp), and Li et al^16^ (up to 5% for 300 kVp). Our average difference was −0.1 ± 2.0% for 250 kVp, consistent with that reported by Xiong et al^31^ of −0.7 ± 1.0% for their 150-kVp beam.

In our study, PDD increased with increases in energy or applicator size (Figures 3 and 4), as expected.^31^^,^^32^ Our circular applicator data agreed better with BJR-25 data than did the square data, which may be attributed to conversion to equivalent squares from the BJR-25 data. The greatest differences between our measurements and BJR-25 data were observed for 250 kVp with the 10 × 10 cm^2^ open field, in which the film measurements were lower by about −4% compared with BJR-25 data, which may be due to interpolations with respect to beam quality or FSD. Sources of uncertainties in our measurement include setup uncertainties, output fluctuations, variation in mass energy absorption coefficient with depth, beam quality specification uncertainties, and film dosimetry uncertainties. However, differences between measurements and BJR-25 data could arise from not only measurement uncertainties but also BJR-25 data uncertainties, as noted earlier. The BJR-25 data are an aggregate from a vast range of beams with varying internal/external filtration obtained from many centres with machines by different manufacturers, and they were older model machines. The specified kVp/HVL may not fully represent the characteristics of each beam’s spectra. Moreover, a range of different detectors, phantom materials, and measurement methodologies were used for PDD measurements at the various centres. Furthermore, the BJR-25 values used for comparison were obtained by interpolations with respect to HVL, FSD, field size, and conversion from circular to square fields when applicable. Each component adds to the overall uncertainty of the BJR-25 values.

BJR-25 recommended “judicious use” of their data, especially in centres without suitable dosimetric equipment or experienced staff, but centres with advanced dosimetry capabilities can perform direct measurements and check for any departure of their results from BJR-25 data.^9^ Similarly, AAPM TG-61 stated that BJR-25 data “shall be used” if an appropriate detector for PDD measurements could not be identified.^10^ A recent survey conducted in the United Kingdom showed that most centres (57%) rely on BJR-25 PDD data for their treatment planning, instead of performing local PDD measurements.^17^ However, considering the limitations of the BJR-25 data, some use BJR-25 data only as a secondary comparison dataset and strongly advocate for PDD measurements of each site-specific energy/applicator combination.^20^^,^^21^ Among the limited published kilovoltage x-ray PDD data (measurements or Monte Carlo simulations) with BJR-25 data comparisons,^12^^,^^13^^,^^16^^,^^18–21^^,^^31^ there exists a wide spectrum of kilovoltage x-ray units, beam qualities, FSD, applicator types/sizes, detectors, and phantom materials used, each with its own nuances, limitations, and practical problems in the measurement methodologies. Given these limitations, for the purpose of verifying site-specific measurements, we believe the heavily scrutinized BJR-25 data are appropriate as the benchmark and the comparisons can be further supplemented with PDD data from other publications such as ours.

Dating back to a basal cell cancer treatment in 1899,^33^^,^^34^ dermatologists have been using radiotherapy for the treatment of skin cancers and other dermatologic indications.^3^^,^^35^ In the mid-1980s, the use of surgical techniques increased and some kilovoltage x-ray therapy units were discontinued, resulting in decreased use of radiotherapy by dermatologists^5^^,^^6^ and elimination of radiotherapy from the training of dermatology residency programs.^8^ Nonetheless, in recent years, with the reintroduction of modern kilovoltage x-ray therapy units that are easier to use, there has been a resurgence in the use of radiotherapy for the management of nonmelanoma skin cancer.^3^^,^^5^^,^^6^^,^^8^ This is especially promising for patients with high-risk disease or with the cancer location in a cosmetically sensitive region or those with contraindications for surgery.^3^ Although some dermatologists refer to hospital-based radiation oncologists, many dermatologists’ offices now offer kilovoltage x-ray treatments^36^ as a cost-effective treatment option.^6^^,^^8^ Use of radiotherapy in dermatologists’ offices is likely to continue increasing. We speculate that more kilovoltage x-ray therapy units will be commissioned. To ensure accurate dose delivery to the patients, the new unit’s beam PDD should be compared with published values. Our results show that BJR-25 data are still a valid benchmark, and the PDD data in the current study can be reproduced to serve as a comparison dataset as well. Moreover, recent education for dermatologists may have some gaps with the use of radiotherapy,^6^ warranting additional practical training in radiotherapy in dermatology residency programs.^5^^,^^6^^,^^35^ The figures presented here can aid clinicians’ general understanding of the influence of technical parameters on dose at depth, empowering clinicians to deliver radiotherapy safely and effectively.

A limitation of the current study was that only three specific energies were characterized. These three beam qualities were commissioned at our institution based on our physicians’ experience and preference. Although our measurements were performed on an Xstrahl 300 kilovoltage x-ray therapy unit, the close agreement with BJR-25 data suggests that similar PDD curves can be expected for other kilovoltage x-ray units as well. Generally, published PDD data in the kilovoltage energy range are limited, generated from various kilovoltage x-ray therapy units with varying beam qualities, FSD, and field sizes.^11–13^^,^^15^^,^^16^^,^^18–21^^,^^29^^,^^31^^,^^32^ Our film-based measurements add to this literature to serve as a basis for comparison, enabling others to find datasets that best coincide with their specific beam qualities, FSD, and applicators. We also provide high spatial resolution data, compared with most other measurements performed using point detectors.

In conclusion, we presented PDD data for various energies and field sizes for an Xstrahl 300 kilovoltage x-ray therapy unit. Centres wishing to compare the PDD measurements of their kilovoltage beams can refer to our PDD data, as well as that reported in BJR-25, which we confirmed can still serve as a valid comparison.
